# Use of Traditional, Complementary and Integrative Medicine During the COVID-19 Pandemic: A Systematic Review and Meta-Analysis

**DOI:** 10.3389/fmed.2022.884573

**Published:** 2022-05-09

**Authors:** Tae-Hun Kim, Jung Won Kang, Sae-Rom Jeon, Lin Ang, Hye Won Lee, Myeong Soo Lee

**Affiliations:** ^1^Korean Medicine Clinical Trial Center, Korean Medicine Hospital, Kyung Hee University, Seoul, South Korea; ^2^Department of Clinical Korean Medicine, Graduate School, Kyung Hee University, Seoul, South Korea; ^3^Department of Acupuncture and Moxibustion, College of Korean Medicine, Kyung Hee University, Seoul, South Korea; ^4^KM Science Research Division, Korea Institute of Oriental Medicine, Daejeon, South Korea; ^5^Korean Convergence Medicine, University of Science and Technology, Daejeon, South Korea; ^6^KM Convergence Research Division, Korea Institute of Oriental Medicine, Daejeon, South Korea

**Keywords:** prevalence of usage, complementary and alternative medicine, systematic review, meta-analysis, STROBE statement, Traditional Complementary and Integrative Medicine, TCIM

## Abstract

**Background:**

Traditional, Complementary and Integrative Medicine (TCIM) has been reported to use for symptom management of coronavirus disease 2019 (COVID-19). The objective of this review was to identify the overall usage prevalence of TCIM interventions for COVID-19.

**Methods:**

Surveys on the general population and observational studies on the COVID-19 patient chart review were located in the search of PubMed, EMBASE and Cochrane Central Register of Controlled Trials databases in September 2021. Observational studies, such as cross-sectional studies, surveys, cohort studies and hospital-based patient case reviews, published in any language, reporting the usage of TCIM in the patients with COVID-19 or the general population during the COVID-19 pandemic were included in this review. Data screening and extraction were performed independently by two reviewers. The reporting quality of the included studies was assessed with the STrengthening the Reporting of OBservational studies in Epidemiology (STROBE) statement. To conduct a meta-analysis of the usage prevalence of various TCIM interventions, the effect size of the proportion for each intervention was calculated with the inverse variance method. The main outcome was usage prevalence of TCIM interventions among patients with COVID-19 or the general population during the COVID-19 pandemic.

**Results:**

A total of 62 studies were included in this review. The overall TCIM usage prevalence was estimated to be 0.64 (95% CI 0.54–0.73). The overall prevalence did not differ between the population-based survey (0.65, 95% CI 0.48–0.81) and the hospital-based patient case review (0.63, 95% CI 0.52–0.73). Statistical heterogeneity and comparatively low quality in reporting were observed, which should be cautiously considered when interpreting the results.

**Conclusion:**

Various TCIM interventions were reported to be used with comparatively high frequency. Future international collaborative research might overcome the main limitation of this study, i.e., the heterogeneity of the included data.

**Systematic Review Registration:**

https://www.crd.york.ac.uk/prospero/display_record.php?RecordID=278452, identifier: CRD42021278452.

## Introduction

Coronavirus disease 2019 (COVID-19) is an acute upper respiratory tract infection which is caused by severe acute respiratory syndrome coronavirus 2 (SARS-CoV-2) newly identified in 2019 (1). Since the World Health Organization (WHO) declared a pandemic in March 2020, several variants have been appearing, and the pandemic continues. Treatment strategies have been gradually established based on accumulated clinical evidence for the acute treatment of COVID-19. In addition to the basic management principle that prioritizes the prevention of complications such as thrombosis, along with adequate oxygenation and hemodynamic support, antiviral agents such as remdesivir, antibody therapy and drug treatments such as dexamethasone are being used in clinical practice (2, 3). However, since knowledge about this disease is still incomplete and new mutated viruses continue to emerge, uncertainty is rising about the basis of treatment from a medical point of view. Additionally, symptoms that persist after the acute manifestation of COVID-19, called long COVID-19, should not be overlooked (4). Patients with severe clinical presentation of COVID-19 are likely to experience long-term respiratory system dysfunction or sequelae of complications, but the real problem is that a significant number of mild infections or asymptomatic COVID-19 patients have been presenting with long COVID symptoms for a long time (5). In addition, long COVID patients complain of symptoms of various spectra, such as fatigue, cognitive decline, respiratory difficulty, joint pain, loss of taste/smell and hair loss (4). From this perspective, a multidisciplinary approach is required for the prevention and management of COVID-19 from the acute stage through long COVID (3, 4).

Since the outbreak of COVID, Traditional, Complementary and Alternative Medicine (TCIM) has been adopted as an alternative strategy for the prevention and treatment of COVID-19 as the entire medical capacity of a country is mobilized at a national level to promote immunity and to protect against viral infection at both the national and individual levels. Clinical practice guidelines (CPGs) or therapeutic protocols for the management of COVID-19 have been published in various fields of TCIM, such as Traditional Chinese Medicine (TCM) (6, 7), Korean Medicine (8), and Ayurveda (9). In addition, according to a survey conducted on patients in quarantine in India, 25% of patients responded that they had experiences with TCIM-related products or home remedies (10). Judging from these data and recently published bibliometric studies (11–13). TCIM interventions are being used quite actively for the management of COVID-19 worldwide, although usage status might variations across countries might depending on the country's medical system. Considering each country's situation, reviewing the utilization prevalence of overall TCIM interventions by country and the utilization status of each intervention may provide insight into the impact of TCIM on global health in the COVID-19 pandemic period.

Therefore, we investigated the prevalence of TCIM use to prevent and treat COVID-19 around the world, identified the most frequently used specific TCIM treatments per country and suggested the overall proportion of CAM use worldwide to treat COVID-19 using systematic review methods.

## Methods

This was a systematic review (SR) for observational studies that assessed the usage status of TCIM interventions worldwide. We located surveys of the general population to assess the usage status of TCIM interventions and hospital-based COVID patients' case review studies through electronic database searches. Overall and individual usage prevalence of diverse TCIM interventions were estimated through meta-analysis. This review protocol was registered in PROSPERO (https://www.crd.york.ac.uk/prospero/display_record.php?RecordID=278452).

Review questions

1. How frequent are TCIM interventions used in the general population and COVID-19 patients worldwide?
2. How much is the difference in usage prevalence between different TCIM interventions?

### Inclusion Criteria

#### Population

We did not impose any limitations on the population if the study assessed the usage prevalence of TCIM interventions during the COVID-19 pandemic. Both healthy individuals and COVID-19 patients were included in this review.

#### Intervention

In this review, we allowed any type of TCIM intervention based on the definition of the U.S. National Center for Complementary and Integrative Health (14). Interventions included nutrition (e.g., special diets, dietary supplements, herbs, probiotics, microbial-based therapies and botanical drugs), psychological treatment (e.g., meditation, hypnosis, music therapies, relaxation therapies, qigong, hypnotherapy, Feldenkrais method, Alexander technique, Pilates, Rolfing Structural Integration, and Trager psychophysical integration), physical therapies (e.g., acupuncture, massage and spinal manipulation), combinations such as psychological and physical methods (e.g., yoga, tai chi, dance therapies and some forms of art therapy), psychological and nutritional combinations (e.g., mindful eating), chiropractic and osteopathic manipulation or traditional medicine (e.g., Ayurvedic Medicine, Traditional Chinese Medicine, homeopathy, naturopathy and functional medicine).

#### Comparator

We included observational studies, so most studies were not expected to have comparator groups. However, any kind of comparator intervention was allowed.

#### Outcome

Usage prevalence of TCIM interventions among patients with COVID-19 or the general population during the COVID-19 pandemic was included.

#### Design

Observational studies, such as cross-sectional studies, surveys, cohort studies and hospital-based patient case reviews, were included in this review.

### Literature Search and Data Extraction

Core databases, including PubMed, EMBASE and Cochrane Central Register of Controlled Trials (CENTRAL), were searched in September 2021. The search strategy was developed with COVID-19-related terms and keywords for TCIM for each database, and the PubMed search strategy was listed in the Supplementary Table 1.

Screening and selection of the studies for inclusion in this review were conducted manually by two authors (THK and SRJ) independently. Any disagreement was arbitrated by the third author (JWK). EndNote 20 (Philadelphia, PA) was used for the screening stage of this review. We uploaded a list of located publications and conducted a screening process with this software. The predefined extraction form included data regarding the type of study, population (healthy individuals or COVID-19 patients), type of TCIM interventions, country, time point for acquiring study data, information on the study population (age and sex), purpose of the usage of TCIM interventions and numbers of patients in hospital-based case reviews and survey respondents (numbers in the total population and those who used TCIM interventions).

### Quality Assessment

For this review, observational patient case reviews for COVID-19 patients and surveys for the general population or COVID-19 patients were included. Therefore, items from the STrengthening the Reporting of OBservational studies in Epidemiology (STROBE) statement were selectively used for assessing the reporting quality of the included studies. We evaluated appropriateness of reporting for items including title and abstract, objectives, participants, variable, data sources, bias, study size, statistical methods, number of participants in each stage of the result section, result analysis, key results, limitations and funding (including conflicts of interest). Each item was evaluated with “A” if all the necessary points were appropriately suggested in the publication of the study and “I” if not. Two authors (THK and SRJ) independently assessed STROBE items and discussed them until they reached agreement.

### Data Synthesis

To conduct a meta-analysis of the usage prevalence of various TCIM interventions, the effect size of the proportion for each intervention was calculated with the inverse variance method. When calculating the estimated proportion of TCIM usage, we used double arcsine transformation and back transformation methods, because some studies showed extreme proportions, such as close 0 or 1, which meant that the dataset was skewed and not normally distributed (15). A random effects model was adopted to calculate summary effect estimates of usage prevalence, because there could be potential clinical heterogeneity in the study methods and study population. Based on the study types (survey vs. hospital-based COVID patient case review), intervention types, study population (general population vs. hospitalized patients) and the country where the intervention was used, subgroup analysis was conducted. *I*^2^ statistics were used to assess statistical heterogeneity. In the subgroup analysis, the *R*^2^ index was calculated to quantify the amount of variance or how much of the total variance in the meta-analysis could be explained by the suspicious effect modifier (16). Publication bias was assessed through visual evaluation of funnel plots and Egger's test. The package “meta” and the function “metaprop” in R (ver 4.0.2) were used for meta-analysis of proportions in this study. The overall usage prevalence of TCIM interventions in each country was presented in the form of a world map using the “ggplot2” package.

## Results

### Summary of the Included Studies

**Figure 1:**
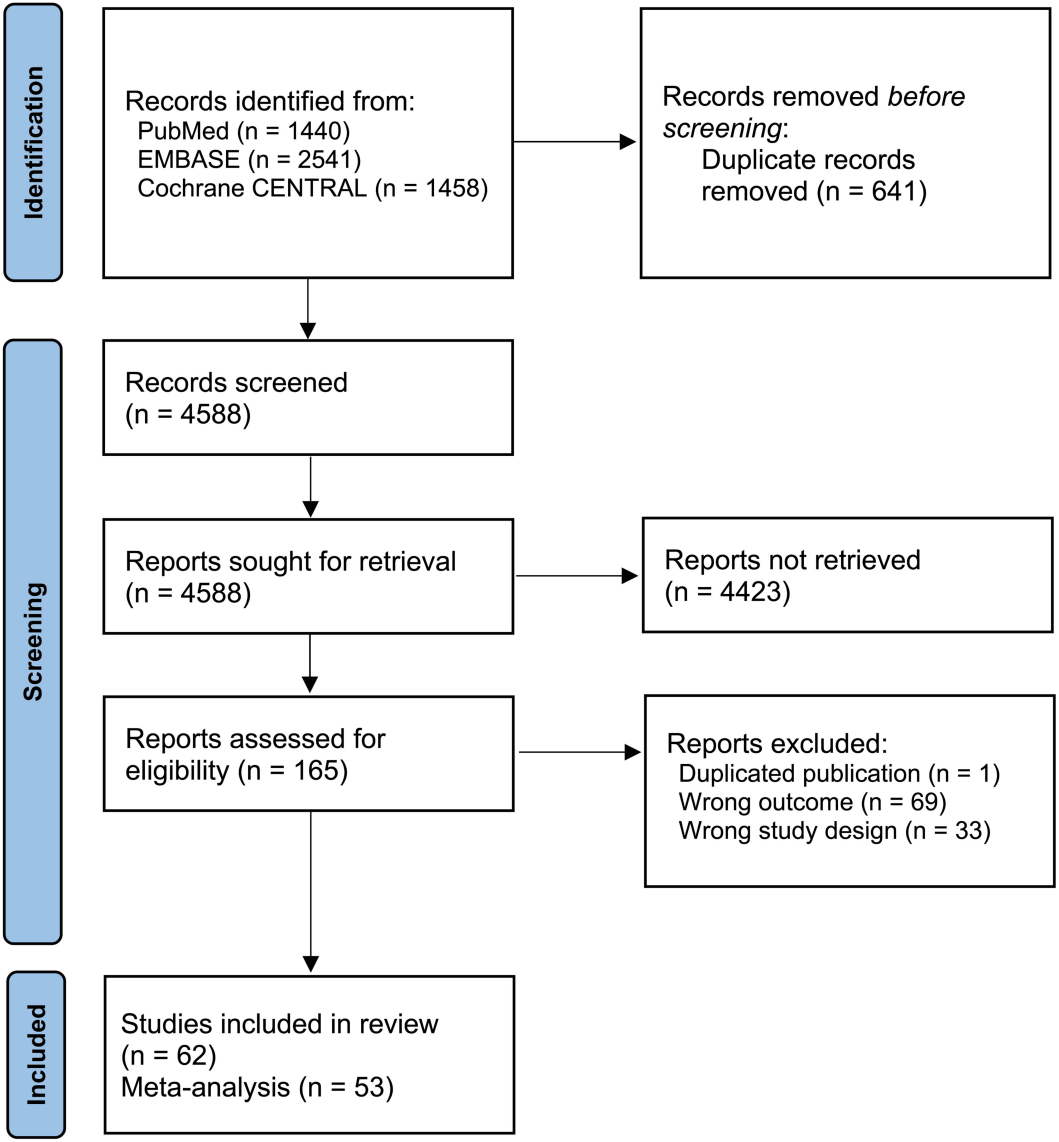
Study flow diagram.

From the electronic database search, a total of 62 studies were enrolled in this review (Figure 1) (10, 17–77). Thirty-nine studies were population-based surveys, and twenty-three studies were hospital-based COVID-19 patient case reviews. One study was a survey that included patient data from two regions, Hong Kong and mainland China, separately, so we analyzed the data separately (71). Twenty-two studies were conducted in China (27, 31–34, 37, 39, 40, 44, 47, 55, 62–65, 67, 69–73, 75–77), six in Saudi Arabia (18, 21–23, 25, 26), five in India (10, 41, 46, 60, 61), three in the United States (35, 42, 45) and three in Turkey (38, 49, 66), and these were the most frequent countries included in this review. Among the population-based survey studies, thirty-one were conducted through online surveys only (17, 18, 20, 22, 23, 25, 26, 28–31, 35, 42, 43, 45, 48–54, 57–62, 66, 68, 74), one was an in-person interview (56), three were telephone interviews (10, 21, 38), and two were both in-person and online surveys (19, 36). Among the included studies, usage status of the interventions, including TCM (*n* = 22) (27, 32–34, 37, 39, 40, 44, 47, 55, 63–65, 67, 69–73, 75–77), functional food (supplements) or herbs (*n* = 20) (17–24, 29, 36, 38, 43, 49–52, 57–59), mind-body practice or spiritual practice (*n* = 9) (28, 35, 42, 45, 48, 54, 60, 61, 74), Ayurveda (*n* = 2) (10, 41), homeopathy (*n* = 1) (46) and Ethiopian traditional medicine (*n* = 1) (56) was suggested. Only one study was conducted in 2021 (57). Most of the data for surveys or patient chart reviews were collected during 2020. In approximately half of the studies, TCIM interventions were used for prophylactic purposes (*n* = 28) (17–20, 22, 23, 25, 26, 28, 31, 35, 36, 38, 41, 42, 45, 48–54, 59, 61, 62, 66, 74), and the other half were used for therapeutic purposes (*n* = 27) (21, 24, 27, 32–34, 37, 39, 40, 44, 46, 47, 55, 58, 63–65, 67, 69–73, 75–77) (Table 1).

**Table 1:** Summary of the included studies.

**Study ID**	**Type of study**	**Study population**	**Method for data acquisition**	**Type of TCIM intervention**	**Country**	**Time points for data acquisition**	**Sex (male)**	**Sex (female)**	**Age (mean or median with SD or ranges, years)**	**Purpose of TCIM usage**
Abdulateef et al. (17)	Survey	Recovered patients	Online survey for patients recovered from COVID-19	Dietary supplements	Iraq	July 1st, 2020	190	238	Median 33 (15–80)	Prophylactic
Abdullah and Naif Al-Harbi (18)	Survey	General population who used herbal medicine and dietary supplements	Online survey for general population	Herbs and natural products	Saudi Arabia	May–Jul, 2020	235	819	Average 35.1 (18–70)	Prophylactic
Ahmed et al. (19)	Survey	General population	Online Survey or in-person interview on general population	Herbal food and dietary supplements	Bangladesh	Jun–Jul, 2020	750	466	Mean 30.77 (SD 12.1)	Prophylactic
Al-Samydai et al. (20)	Survey	General population	Online survey for general population	Herbs	Jordan	Sep–Oct 2020	128	159	Not reported	Prophylactic
Aldwihi et al. (21)	Survey	Recovered patients	Telephone survey interview on the recovered patients	Herbs and dietary supplements	Saudi Arabia	Aug–Oct 2020	418	320	Average 36.5 (11.9)	Therapeutic
Alfawaz et al. (22)	Survey	General population	Online survey for general population	Dietary supplements	Saudi Arabia	May–Jun 2020	450	508	Average 36.7 (13.8)	Prophylactic
Alnajrany et al. (23)	Survey	General population	Online survey for general population	Natural products	Saudi Arabia	Mar–Aug 2020	605	868	Not reported	Prophylactic
Alonso-Castro et al. (24)	Survey	General population with anxiety and depression in adults	Not reported	Herbal products	Mexico	Mar–Jun 2020	893	1,207	Average 32.08 (13.57)	Therapeutic
Alqahtani et al. (25)	Survey	General population	Online survey for general population	Vitamins, herbs, and traditional medicine	Saudi Arabia	Nov 2020	3,278	2204	Not reported	Prophylactic
Alyami et al. (26)	Survey	General population	Online survey for general population	Herbal product and food supplements	Saudi Arabia	May–Jun 2020	2,258	3,000	Not reported	Prophylactic
An et al. (27)	Cross sectional study	Convalescents of COVID-19	Hospital-based COVID-19 patient case reviews	TCM decoctions	China	Feb–May 2020	Not reported	Not reported	Not reported	Therapeutic
Ashiq et al. (28)	Survey	General population	Online survey for general population	Exercise, yoga or walk	Pakistan	Mar–Apr 2020	147	169	Not reported	Prophylactic
Azhar et al. (29)	Survey	General population	Online survey for general population	Herbal medication and dietary supplements	Pakistan	2020	91	179	Not reported	Not reported
Barnes et al. (30)	Survey	Patients with irritable bowel syndrome	Online survey for irritable bowel syndrome	Exercise, probiotics, meditation, mindfulness, acupuncture, prayer, TCM and Homeopathy	Australia	May–Jul 2020	101	143	Median 46 (IQR 35–57)	Prophylactic or therapeutic
Ben-Ezra et al. (31)	Survey	General population under quarantine due to COVID-19	Online survey for general population	Vitamins and TCM medicines	China	Apr 2020	527	607	Average 30.99 (6.82)	Prophylactic
Cen et al. (32)	Cross sectional study	COVID patients	Hospital-based COVID-19 patient case reviews	TCM medication (Lianhua Qingwen)	China	Feb 2020	493	514	Not reported	Therapeutic
Charan et al. (10)	Survey	COVID-19 patients without symptoms	Telephone survey interview in isolation center	Ayurveda, herbal products	India	2020	Not reported	Not reported	Not reported	Prophylactic or therapeutic
Chen et al. (33)	Cross sectional study	Severe COVID-19 patient	Hospital-based COVID-19 patient case reviews	TCM patient medications	China	2020	296	366	Average 60 (47–70)	Therapeutic
Cheng et al. (34)	Survey	COVID patients	Survey not detailed information in shelter hospital	TCM medications	China	Feb–Mar 2020	67	49	Average 44 (22–57)	Therapeutic
Chrisinger et al. (35)	Survey	General population	Online Survey for general population in a community-based cohort	Contemplative practice behaviors (mindfulness, compassion practices)	United States	Apr 2020	176	724	Not reported	Prophylactic
de los Angeles et al. (36)	Survey	General population	Online Survey or in-person interview on general population	Herbal products	Ecuador	Jan–Mar 2020	350	479	Not reported	Prophylactic
Du et al. (37)	Cross sectional study	Pediatric COVID patients	Hospital-based COVID-19 patient case reviews	TCM medication	China	Jan–Feb 2020	120	62	Median 6 (0.01–15)	Therapeutic
Erdem et al. (38)	Survey	Cancer patients	Telephone survey interview on outpatient community-based oncology clinic	Dietary supplement	Turkey	Apr 2020	101	199	Average 57.39 (19–92)	Prophylactic
Feng 2020a et al. (39)	Cross sectional study	COVID patients	Hospital-based COVID-19 patient case reviews	TCM medication	China	Feb–Mar 2020	65	69	Median 45 (33–56)	Therapeutic
Feng 2020b et al. (40)	Cross sectional study	COVID patients with severe symptom	Hospital-based COVID-19 patient case reviews	TCM medication	China	Jan–Feb 2020	71	43	Average 63.96 (13.41)	Therapeutic
Francis et al. (41)	Survey	Students	Not reported	Ayurvedic foods	India	Not reported	416	409	Not reported	Prophylactic
Green et al. (42)	Survey	General population using meditation app	Online survey for general population using meditation online app	Meditation	United States	Apr–May 2020	1,147	6,129	Average 47 (13.8)	Prophylactic
Hamdani et al. (43)	Survey	General population	Online survey for general population	Herbal medication	Algeria	Not reported	230	370	Average 36	Prophylactic or therapeutic
He et al. (44)	Cross sectional study	COVID patients (children)	Hospital-based COVID-19 patient case reviews	TCM	China	Jan–Jun 2020	18	17	Average 7.1 (4.2)	Therapeutic
Hellem et al. (45)	Survey	General population	Online survey with email and social media for general population	Mind-body practice, physical exercise	United States	Apr–Jun 2020	29	304	Average 49.7 (16.1)	Prophylactic
Jethani et al. (46)	Cross sectional study	COVID patients	hospital-based COVID-19 patient case reviews	Homeopathy	India	Apr–Jun 2020	142	54	Average 38.9 (16.3)	Therapeutic
Ji et al. (47)	Cross sectional study	COVID patients with stroke	Hospital-based COVID-19 patient case reviews	TCM	China	Feb–May 2020	17	10	Average 66.4 (12.1)	Therapeutic
Jimenez et al. (48)	Survey	General population	Online survey for general population	Mind-body practice	Spain	Not reported	61	348	Not reported	Prophylactic
Kamarli et al. (49)	Survey	General population	Online survey for dietitians	Dietary supplements, functional foods, herbal medicine	Turkey	May–Jun 2020	65	485	Average 30.6 (9.1)	Prophylactic
Karbownik et al. (50)	Survey	General population	Online survey for general population	Dietary supplements	Poland	Mar–May 2020	65	369	Average 36.4 (13.9)	Prophylactic
Khadka et al. (51)	Survey	General population	Online survey for general population	Medicinal plants	Nepal	Jun–Jul 2020	471	303	Not reported	Prophylactic
Kristiandi et al. (52)	Survey	General population (undergraduate student)	Online survey for undergraduate student	Dietary supplements	Indonesia	Jun 2020	845	5,079	Not reported	Prophylactic
Lam et al. (53)	Survey	General population	Online survey for general population	Dietary supplement, TCM medication, acupuncture, massage, aromatherapy, yoga, qigong and moxibustion	China	Nov–Dec 2020	233	399	Not reported	Prophylactic
Lenaerts et al. (54)	Survey	General population	Online survey for general population	Nature visits	Belgium	Not reported	3,568	7,742	Not reported	Prophylactic
Ma et al. (55)	Cross sectional study	COVID patients after acute admission treatments	Hospital-based COVID-19 patient case reviews	TCM	China	Feb 2020	348	361	Average 45.15 (12.64)	Therapeutic
Mamo et al. (56)	Survey	General population	In-person survey interview on general population	Traditional medicine	Ethiopia	May–Jun 2020	547	307	Average 34.12 (18–89)	Prophylactic or therapeutic
Mohsen et al. (57)	Survey	General population	Online survey for general population	Dietary supplement	Lebanon	Jan–Feb 2021	1,449	1,522	Average 29.47 (11.4)	Prophylactic or therapeutic
Nguyen et al. (58)	Survey	General population	Online survey for general population	Herbal medicine	Vietnam	Sep–Oct 2020	180	328	Average 26.8 years (18–68)	Therapeutic
Panagiotakos et al. (59)	Survey	General population	Online survey for general population	Dietary supplement	Greece	Dec 2020	912	1,346	Median 35 years (31–45)	Prophylactic
Parimala et al. (60)	Survey	General population	Online survey for general population	Yoga	India	Mar–May 2020	Not reported	Not reported	Average 42.99 years (16–81)	Not reported
Sahni et al. (61)	Survey	General population	Online survey for general population	Yoga and spiritual practice	India	Apr–Jun 2020	416	223	Not reported	Prophylactic
Shi et al. (62)	Survey	General population	Online survey for general population	TCM herbs, Physical exercise	China	Feb 2020	569	2,082	Average 35.91 year (10.95)	Prophylactic
Shu et al. (63)	Cross sectional study	COVID patients in hospital	Hospital-based COVID-19 patient case reviews	TCM herbal prescriptions	China	Jan–Mar 2020	135	158	Average 57.1 year (15.6)	Therapeutic
Sun et al. (64)	Cross sectional study	COVID patients in hospital	Hospital-based COVID-19 patient case reviews	TCM	China	Jan–Apr 2020	84	81	Average 55 years (42–66)	Therapeutic
Sun et al. (65)	Cross sectional study	COVID patients in hospital	Hospital-based COVID-19 patient case reviews	TCM patent medications	China	Jan–Mar 2020	148	134	Average 67 years (59–74)	Therapeutic
Teke et al. (66)	Survey	Healthcare professionals	Online survey for healthcare professionals	TCM, dietary supplements, religious practice	Turkey	Apr 2020	462	98	Average 30.88 years (7.68)	Prophylactic
Tian et al. (67)	Cross sectional study	Severe COVID-19 patient	Hospital-based COVID-19 patient case reviews	TCM	China	Not reported	17	20	Average 44.3 years (1.67)	Therapeutic
Van der Werf et al. (68)	Survey	General population	Online survey for general population	TCIM	The Netherlands	May 2020	495	509	Not reported	Prophylactic or therapeutic
Wan et al. (69)	Cross sectional study	COVID patients in hospital	Hospital-based COVID-19 patient case reviews	TCM	China	Jan–Feb 2020	72	63	Average 47 years (36–56)	Therapeutic
Wang et al. (70)	Cross sectional study	COVID patients in hospital	Hospital-based COVID-19 patient case reviews	TCM	China	Jan–Feb 2020	105	94	Average 46.3 years (16.4)	Therapeutic
Wong et al. (71)^*^	Cross sectional study	COVID patients in hospital	Hospital-based COVID-19 patient case reviews	TCM	China	Jan–Feb 2020	839	3,932	Not reported	Therapeutic
Wong (71)^*^	Cross sectional study	COVID patients in hospital	Hospital-based COVID-19 patient case reviews	TCM	China	Jan–Feb 2020	43	605	Not reported	Therapeutic
Wu et al. (72)	Cross sectional study	COVID patients in hospital	Hospital-based COVID-19 patient case reviews	TCM	China	Jan–Feb 2020	39	41	Average 46.1 years (15.42)	Therapeutic
Yan et al. (73)	Cross sectional study	COVID patients in hospital	Online survey for general population	TCM medication	China	Jan–Jun 2020	122	96	Average 42.9 years (32.0–52.3)	Therapeutic
Zaworski et al. (74)	Survey	General population	Hospital-based COVID-19 patient case reviews	Physical activity	Poland	Apr 2020	197	491	Average 28.61 years (9.5)	Prophylactic
Zhang et al. (75)	Cross sectional study	COVID patients in hospital	Hospital-based COVID-19 patient case reviews	TCM medication	China	Jan–Feb 2020	23	30	Average 46.3 years (19.6)	Therapeutic
Zhang et al. (76)	Cross sectional study	COVID patients in hospital	Hospital-based COVID-19 pediatric patient case reviews	TCM medication	China	Jan–Feb 2020	92	80	Average 47.9 years (18.4)	Therapeutic
Zhou et al. (77)	Cross sectional study	Pediatric COVID patients in hospital	Online survey for patients recovered from COVID-19	TCM medication	China	Jan–Feb 2020	2	5	Median 3 years	Therapeutic

* *This study included two data sets of different two areas, Hong Kong and mainland China in a study*.

*TCM, Traditional Chinese Medicine; TCIM, Traditional Complementary and Integrative Medicine; SD, standard deviation; IQR, interquartile range*.

### Reporting Quality of the Included Studies

When assessing reporting quality with STROBE statement items, most studies did not appropriately address all the necessary contents in the publications. The most poorly reported items are variables (including effect modifiers and confounders), potential bias, sample size calculation, statistical methods with adjustments of potential effect modifiers and detailed information on excluded participant numbers at each stage of the study (Supplementary Table 2).

### Overall Estimated Prevalence of TCIM Usage

**Figure 2:**
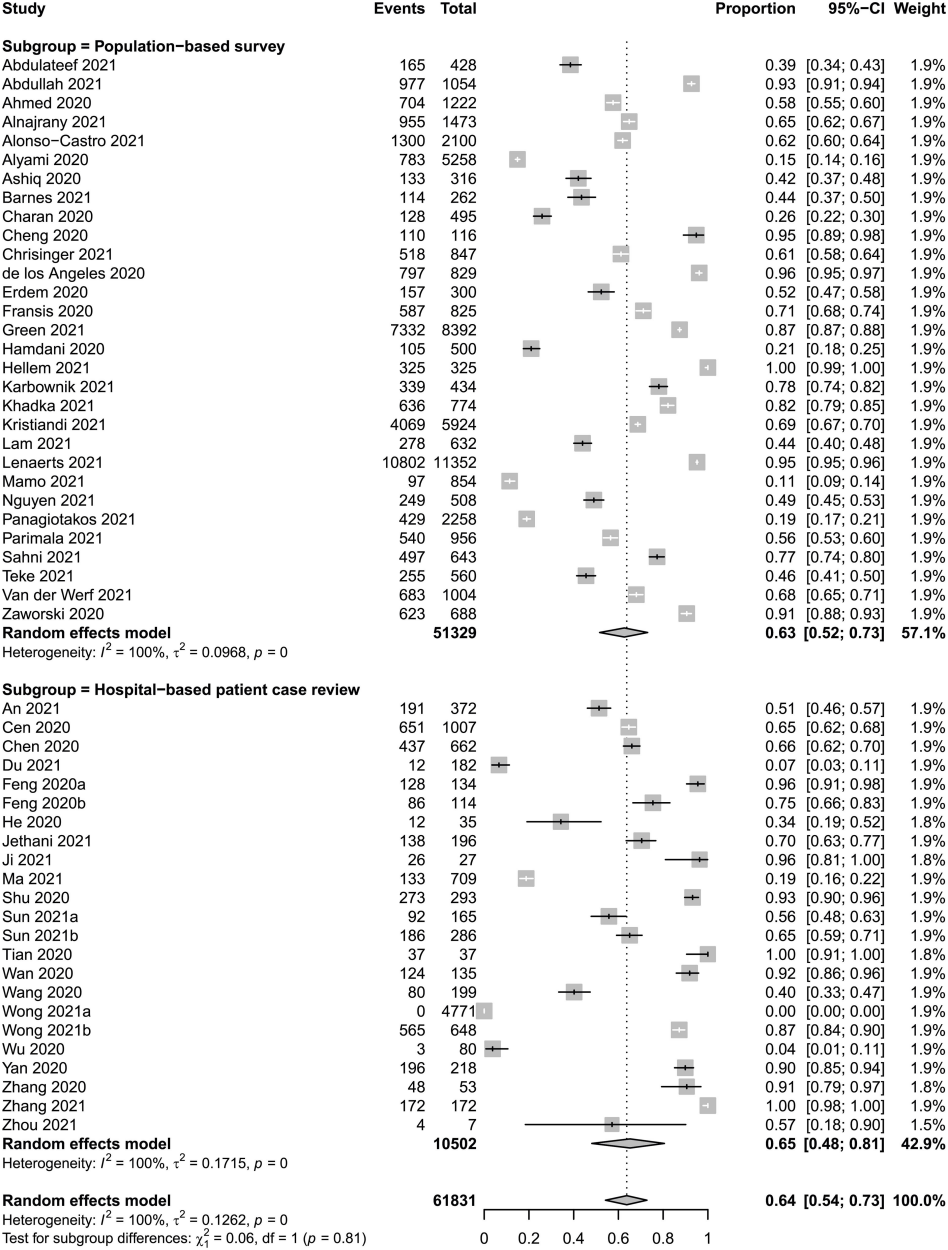
Meta-analysis of overall usage prevalence of TCIM interventions.

From the included studies, 53 studies (*n* = 61,831) suggested a total number of respondents or patients who used TCIM interventions during the COVID pandemic period. The overall pooled prevalence of TCIM usage was estimated to be 0.64 (95% CI 0.54–0.73). The *I*^2^ statistic was 99.88%, which implied severe statistical heterogeneity among the included studies (Figure 2). In screening for outliers by evaluating the studentized residuals of the included studies, one study (71) was identified as a potential outlier (*z* = −2.6827, Supplementary Figure 1). When this study was excluded, the pooled prevalence was estimated to be 0.65 (95% CI 0.56–0.74). In the population-based survey, the overall prevalence of TCIM usage was 0.63 (95% CI 0.52–0.73), which did not show a severe difference when compared with findings from the hospital-based patient case review (overall prevalence 0.65, 95% CI 0.48–0.81, Figure 2).

To analyze statistical heterogeneity among the included studies, subgroup analysis was conducted based on potential effect modifiers, including the type of studies (population-based survey vs. hospital-based COVID-19 patient case reviews), study population (general population vs. COVID-19 patients) and country of the study population (China vs. other countries). The *R*^2^ score for a potential effect modifier and the type of study was estimated to be 0%, which indicated that study type was not a strong effect modifier in this review. The study population (*R*^2^ score = 0%, Supplementary Figure 2) and country (*R*^2^ score = 1.03%, Supplementary Figure 3) could not explain the heterogeneity. In addition, a meta-regression test for the sample size of the included studies did not suggest that study size was an effect modifier (intercept: 0, *P*-value: 0.9108).

When comparing prevalence between countries, the country with the highest usage proportion of TCIM interventions was Ecuador (estimated proportion 0.9614, 95% CI 0.9471–0.9735), and Ethiopia showed the lowest proportion (0.1136, 95% CI 0.0931–0.1358, Table 2). There were no definitive regional trends in the usage proportion of TCIM interventions visually observed from the world map of proportion distribution (Supplementary Figure 4).

**Table 2:** Estimated proportion of TCIM usage in each country.

**Country**	**Proportion**	**95% confidence intervals**	**Number of studies**
Ecuador	0.9614	0.9471–0.9735	1
Belgium	0.9516	0.9475–0.9554	1
United States	0.8796	0.5655–1.0000	3
Poland	0.8491	0.7086–0.9492	2
Nepal	0.8217	0.7939–0.8479	1
China	0.6571	0.4896–0.8071	24
Indonesia	0.6869	0.6750–0.6986	1
Netherlands	0.6803	0.6511–0.7099	1
Mexico	0.619	0.5982–0.6397	1
India	0.6051	0.4168–0.7785	5
Saudi Arabia	0.5902	0.1261–0.9675	3
Bangladesh	0.5761	0.5483–0.6037	1
Vietnam	0.4902	0.4467–0.5337	1
Turkey	0.4864	0.4203–0.5528	2
Australia	0.4351	0.3755–0.4956	1
Pakistan	0.4209	0.3669–0.4758	1
Iraq	0.3855	0.3399–0.4322	1
Algeria	0.21	0.1754–0.2469	1
Greece	0.19	0.1741–0.2064	1
Ethiopia	0.1136	0.0931–0.1358	1

*TCIM, Traditional Complementary and Integrative Medicine*.

### Usage Prevalence of Individual Types of TCIM Interventions

Twenty-five studies assessed the usage prevalence of TCM medication, and the synthesized proportion was estimated to be 0.62 (95% CI 0.45–0.78), which showed severe statistical heterogeneity (*I*^2^ = 100%, Supplementary Figure 5A). The usage proportions of Ayurveda (*n* = 2) and homeopathy (*n* = 2) were 0.44 (95% CI 0.04–0.91, Supplementary Figure 5B) and 0.30 (95% CI 0.00–0.97, Supplementary Figure 5C). The usage prevalence of yoga was suggested in 7 studies, and the estimated proportion was 0.53 (95% CI 0.27–0.78, Supplementary Figure 5D). Acupuncture was assessed in 3 studies, and the estimated prevalence was 0.20 (95% CI 0.00–0.58, Supplementary Figure 5E). Physical exercise was evaluated in 4 studies, and the estimated prevalence was 0.70 (95% CI 0.33–0.96, Supplementary Figure 5F). The proportion of dietary supplements, herbs or natural products used was estimated to be 0.58 (95% CI 0.42–0.73, Supplementary Figure 5G) from the meta-analysis of 14 studies. The usage prevalence of spiritual therapy (*n* = 3) and massage (*n* = 2) were 0.24 (95% CI 0.01–0.65, Supplementary Figure 5H) and 0.28 (95% CI 0.00–0.87, Supplementary Figure 5I), respectively.

### Publication Bias

To assess potential publication bias, visual inspection of funnel plots was conducted, and no obvious asymmetry was observed (Supplementary Figure 6). Egger's test results suggested that there was no significant publication bias (*P* = 0.6856).

## Discussion

From 62 studies, the overall prevalence of TCIM usage during the COVID-19 pandemic was estimated to be 0.64 (95% CI 0.54–0.73), which also showed severe statistical heterogeneity and poor reporting quality. When comparing the rates of TCIM use across countries, the estimated proportion showed very large differences from 0.6914 (95% CI 0.9471–0.9735) in Ecuador to 0.1136 (95% CI 0.0931 to 0.1358) in Ethiopia. Study types for data acquisition did not affect the overall prevalence of TCIM usage 0.63 (95% CI 0.52–0.73) in the population-based survey and 0.65 (95% CI 0.48–0.81) in the hospital-based patient case review (*R*^2^ index = 0%). Although we explored potential effect modifiers for assessing statistical heterogeneity of this review, we failed to identify any meaningful reasons. Type of studies, study population and country of the population could not explain the considerable statistical heterogeneity of this study result.

From this review, it was found that TCIM interventions have been used for preventive and therapeutic purposes. Why do people use TCIM interventions for COVID-19? Disease burden, previous TCIM experience and perception of TCIM efficacy are well-known determinants for the usage of TCIM interventions for disease management (78, 79). During the COVID-19 pandemic when some underdeveloped countries experienced shortage of medical resources and restricted access to medical institutions, TCIM interventions tended to be accepted as a panacea, and this kind of attitude is based on health-related beliefs and the desire for self-care to improve immunity from the viral infection with a holistic approach (26, 80, 81). China published TCM guidelines for COVID-19, which might be related to the high usage rate of TCM interventions for therapeutic purposes during the pandemic period (7, 82–84). Meanwhile, the prevalence of TCIM usage in Western countries, such as the United States, was as high as that in underdeveloped countries. This might be due to different reasons, including dissatisfaction with the quality of conventional healthcare services (85). In addition, TCIM interventions such as mind-body practice have been used to maintain psychosocial health in many countries during the pandemic (35, 42, 45). Regarding reasons for the use of TCIM interventions during the COVID-19 pandemic, it is necessary to evaluate the underlying reasons by region in future research in detail.

This study has limitations. First, we failed to identify meaningful effect modifiers to explain the statistical heterogeneity. The reasons for using TCIM interventions such as prophylactic purpose or therapeutic purpose might be related to the potential heterogeneity of this review result. Different economic statuses and the medical systems of each country might be closely related to the diverse usage status of TCIM interventions, but these factors could not be assessed due to the limited numbers of included studies. In addition, one of the most powerful suspicious factors is the study population of the included studies. Online surveys, which are the most frequent data acquisition methods for the general population, usually adopt convenient sampling or snowballing sampling methods; these methods are commonly used due to easy execution, but they cannot generate unbiased results due to generalizability issues (86). When looking at the process of online surveys in general, a link to the survey questionnaire is posted through the social network services, and interested people primarily participate in the survey. Since people interested in the interventions are more likely to participate in the survey, it is natural that the proportion of end users is high. As a way to solve this problem, it is necessary to use a survey that captures the entire population of interest or probability sampling methods, so that the overall opinion of the general population of interest can be reflected. Hospital-based COVID patient chart review data could also be biased because most studies were conducted in China, which is one of the few countries with published TCIM CPGs for COVID-19 (84, 87, 88). International cooperative surveys on the usage of TCIM interventions would be helpful to overcome the bias introduced by these limitations. Second, the definition of TCIM interventions varied from study to study, so the estimated usage prevalence derived from the meta-analysis of these studies could be inevitably biased. In addition, various study populations, such as the general population, COVID patients, physicians or practitioners of TCIM interventions, could have different perceptions and experiences of TCIM usage during the COVID-19 response. The initial purpose of this study was to suggest a crude prevalence of TCIM usage, so we did not consider these factors when conducting the meta-analysis, which is a critical limitation of this study. In this study, we did not search non-English DBs including China and Korea where TCIM is one of the main medical systems and many relevant studies are published. Therefore, we cannot be confident about locating all relevant data on this topic. Finally, data from the studies conducted mainly in the first half of 2020, shortly after the outbreak of COVID-19, were included in the analysis. The COVID-19 pandemic has been ongoing for nearly 2 years as of November 2021, and current TCIM usage patterns might be different than the initial patterns. An updated review that includes 2021 data is needed. Currently, an international survey on the prevention of and treatments for COVID-19 has been conducted, which might suggest more reliable data on the prevalence of TCIM interventions used worldwide (89).

In conclusion, various TCIM interventions were reported to be used at a comparatively high frequency, but this result should be interpreted carefully due to the heterogeneity and low reporting quality of the included studies. Future studies need to be updated to include global data through international collaborative research, which might overcome the main limitation of this study, i.e., the heterogeneity of the included data.

## Data Availability Statement

The original contributions presented in the study are included in the article/Supplementary Material, further inquiries can be directed to the corresponding author/s.

## Author Contributions

ML and T-HK: conceptualization and writing—original draft. T-HK and S-RJ: methodology and investigation. T-HK: software and visualization. JK and HL: validation. T-HK and LA: formal analysis. T-HK and HL: resources. JK and LA: data curation. JK, S-RJ, LA, and HL: writing—review and editing. T-HK, ML, and JK: supervision. S-RJ and HL: project administration. ML: funding acquisition. All authors read and approved the final manuscript.

## Funding

This study is funded by Korea Institute of Oriental Medicine (KSN20214115).

## Conflict of Interest

The authors declare that the research was conducted in the absence of any commercial or financial relationships that could be construed as a potential conflict of interest.

## Publisher's Note

All claims expressed in this article are solely those of the authors and do not necessarily represent those of their affiliated organizations, or those of the publisher, the editors and the reviewers. Any product that may be evaluated in this article, or claim that may be made by its manufacturer, is not guaranteed or endorsed by the publisher.
