# Supplementary material for: Use of Traditional, Complementary and Integrative Medicine During the COVID-19 Pandemic: A Systematic Review and Meta-Analysis
Source: Front Med (Lausanne). 2022 May 9;9:884573. doi: 10.3389/fmed.2022.884573 (PMC9125211; doi:10.3389/fmed.2022.884573)

Supplementary Table 1. Search strategies

("COVID-19"[MeSH Terms] OR "post-acute COVID-19 syndrome"[Supplementary Concept] OR "COVID-19 stress syndrome"[Supplementary Concept] OR "COVID-19 post-intensive care syndrome"[Supplementary Concept] OR ("sars cov 2"[MeSH Terms] OR "sars cov 2"[All Fields] OR "covid"[All Fields] OR "COVID-19"[MeSH Terms] OR "COVID-19"[All Fields]) OR (("corona"[All Fields] OR "coronae"[All Fields] OR "coronas"[All Fields]) AND ("virology"[MeSH Subheading] OR "virology"[All Fields] OR "viruses"[All Fields] OR "viruses"[MeSH Terms] OR "virus s"[All Fields] OR "viruse"[All Fields] OR "virus"[All Fields]))) AND ("Complementary Therapies"[MeSH Terms] OR "CAM"[All Fields] OR ("Dietary Supplements"[MeSH Terms] OR ("plants, medicinal"[MeSH Terms] OR "Herbals as Topic"[MeSH Terms]) OR "Probiotics"[MeSH Terms] OR "Vitamins"[MeSH Terms] OR "Minerals"[MeSH Terms]) OR ("Meditation"[MeSH Terms] OR "Hypnosis"[MeSH Terms] OR "Relaxation Therapy"[MeSH Terms] OR "Qigong"[MeSH Terms] OR "Exercise Movement Techniques"[MeSH Terms] OR "Musculoskeletal Manipulations"[MeSH Terms]) OR ("Acupuncture"[MeSH Terms] OR "Acupuncture Therapy"[MeSH Terms] OR "acupuncture, ear"[MeSH Terms] OR "Acupuncture Points"[MeSH Terms] OR "Massage"[MeSH Terms] OR "manipulation, spinal"[MeSH Terms]) OR ("Yoga"[MeSH Terms] OR "Tai Ji"[MeSH Terms] OR "Dance Therapy"[MeSH Terms] OR "Art Therapy"[MeSH Terms]) OR ("Chiropractic"[MeSH Terms] OR "manipulation, chiropractic"[MeSH Terms] OR "manipulation, osteopathic"[MeSH Terms]) OR ("medicine, east asian traditional"[MeSH Terms] OR "medicine, korean traditional"[MeSH Terms] OR "medicine, tibetan traditional"[MeSH Terms] OR "medicine, mongolian traditional"[MeSH Terms] OR "medicine, african traditional"[MeSH Terms] OR "medicine, chinese traditional"[MeSH Terms] OR "medicine, traditional"[MeSH Terms] OR "medicine, ayurvedic"[MeSH Terms] OR "Homeopathy"[MeSH Terms] OR "Naturopathy"[MeSH Terms]) OR ("int j complement altern med"[Journal] OR "evid based complement alternat med"[Journal] OR "bmc complement altern med"[Journal] OR ("complementary"[All Fields] AND "and"[All Fields] AND "alternative"[All Fields] AND "medicine"[All Fields]) OR "complementary and alternative medicine"[All Fields])).

Supplementary Table 2. Reporting quality assessment

Study ID	Title and abstract	Objectives	Participants	Variables (effect modifier, confounders)	Data sources and measurement	Bias	Study size	Statistical methods	Numbers of participants in each stage in the result section	Appropriate analysis in the result section	Key results	Limitations	Funding and COI
Abdulateef 2021(13)	I	A	A	I	A	I	A	I	I	A	A	A	A
Abdullah 2021(14)	A	A	A	I	A	I	A	I	I	A	A	I	A
Ahmed 2020(15)	A	A	A	I	A	I	A	I	I	A	A	A	I
Al-Samydai 2021(16)	I	I	I	I	A	I	I	I	I	I	I	I	A
Aldwihi 2021 (17)	I	I	A	I	A	I	I	I	I	A	A	A	A
Alfawaz 2021(18)	A	A	I	I	I	I	I	A	I	A	A	A	A
Alnajrany 2021(19)	A	A	A	I	I	I	A	A	I	A	A	A	A
Alonso-Castro 2021(20)	I	I	I	I	I	I	A	I	I	I	A	A	A
Alqahtani 2021(21)	A	A	A	I	I	I	A	I	I	A	A	A	A
Alyami 2020(22)	A	A	A	I	A	I	A	I	I	A	A	A	A
An 2021(23)	I	A	A	I	I	I	I	I	I	A	A	I	A
Ashiq 2020(24)	I	A	I	I	I	I	I	I	I	A	A	I	A
Azhar 2021(25)	I	I	I	I	I	I	I	I	I	I	A	I	I
Barnes 2021(26)	I	A	A	I	I	I	I	I	I	I	A	A	I
Ben-Ezra 2020 (27)	I	A	A	I	A	I	I	I	I	A	A	A	A
Cen 2020 (28)	A	A	A	A	A	I	I	A	I	A	A	A	A
Charan 2020 (7)	A	I	A	A	A	I	I	I	A	I	A	A	I
Chen 2020 (29)	A	A	A	A	A	I	I	A	A	A	A	A	A
Cheng 2020 (30)	I	A	A	I	A	I	I	A	I	A	A	I	A
Chrisinger 2021(31)	A	A	A	A	A	I	I	A	A	A	A	A	A
de los Angeles 2020 (32)	I	A	I	I	I	I	A	I	I	I	A	A	A
Du 2021 (33)	I	A	A	I	A	I	I	I	I	A	A	A	A
Erdem 2020 (34)	A	A	A	I	I	I	I	I	I	A	A	I	A
Feng 2020a (35)	I	A	A	I	A	I	I	A	I	A	A	A	A
Feng 2020b (36)	I	A	A	I	A	I	I	A	I	A	A	A	A
Francis 2020 (37)	I	I	I	I	I	I	I	I	I	I	A	I	I
Green 2021 (38)	A	A	I	I	A	I	A	A	A	A	A	A	A
Hamdani 2020 (39)	A	I	I	I	I	I	A	I	I	I	A	A	A
He 2020 (40)	I	I	A	I	I	I	I	I	I	I	A	I	A
Hellem 2021 (41)	A	A	I	I	I	I	I	I	A	A	A	A	A
Jethani 2021 (42)	I	A	A	I	I	I	I	I	I	I	A	A	A
Ji 2021 (43)	A	I	I	I	I	I	I	I	I	A	A	A	A
Jimenez 2020 (44)	I	I	I	I	I	I	I	I	I	I	A	A	A
Kamarli 2021 (45)	I	A	A	I	A	I	A	A	A	A	A	A	A
Karbownik 2021 (46)	I	A	I	I	I	I	I	I	I	A	A	A	A
Khadka 2021 (47)	I	A	I	I	I	I	I	I	I	A	A	A	A
Kristiandi 2021 (48)	I	A	A	I	A	I	I	I	I	A	A	I	I
Lam 2021 (49)	A	A	I	I	I	I	I	A	A	A	A	A	I
Lenaerts 2021 (50)	I	A	I	I	I	I	I	I	I	I	A	A	A
Ma 2021 (51)	I	I	A	I	A	I	I	I	A	I	A	A	A
Mamo 2021 (52)	I	I	I	I	I	I	A	A	I	A	A	A	A
Mohsen 2021 (53)	A	A	I	I	I	I	I	A	I	A	A	A	A
Nguyen 2021 (54)	A	A	I	I	I	I	A	A	I	A	A	A	A
Panagiotakos 2021 (55)	A	A	I	I	I	I	I	A	I	A	A	A	I
Parimala 2021 (56)	A	A	I	I	I	I	I	I	I	I	I	A	A
Sahni 2021 (57)	A	I	A	A	I	I	I	A	I	A	A	A	I
Shi 2021 (58)	A	A	A	A	I	I	I	A	I	A	A	A	A
Shu 2020 (59)	I	I	A	A	A	I	I	A	A	A	A	A	A
Sun 2021a (60)	I	I	A	A	A	I	I	I	I	A	A	A	A
Sun 2021b (61)	A	A	A	A	A	I	I	A	I	A	A	A	A
Teke 2021 (62)	I	I	A	I	I	I	I	I	I	A	A	I	I
Tian 2020 (63)	I	I	A	I	A	I	I	I	I	A	A	A	A

Van der Werf 2021 (64)	A	A	A	A	A	A	I	A	A	A	A	A	A
Wan 2020 (65)	I	I	A	I	A	I	I	A	I	A	A	A	A
Wang 2020 (66)	A	A	A	I	A	I	I	I	I	A	A	A	A
Wong 2021a (67)*	A	I	A	A	A	I	I	A	A	A	A	A	A
Wong 2021b (67)*	I	I	A	I	I	I	I	I	I	A	A	A	A
Wu 2020 (68)	I	I	A	I	A	I	I	A	A	A	A	A	A
Yan 2020 (69)	A	I	A	I	A	I	I	I	I	A	A	A	A
Zaworski 2020 (70)	I	A	I	I	I	I	I	I	I	A	A	A	A
Zhang 2020 (71)	I	I	A	I	A	I	I	I	I	A	A	I	I
Zhang 2021 (72)	I	I	A	I	A	I	I	I	I	A	A	A	A
Zhou 2021 (73)	A	I	A	I	A	I	I	I	I	A	A	I	A

A: items which are reported appropriately; I: items which are reported inappropriately.

Supplementary Figure 1. Test for identifying outlier studies

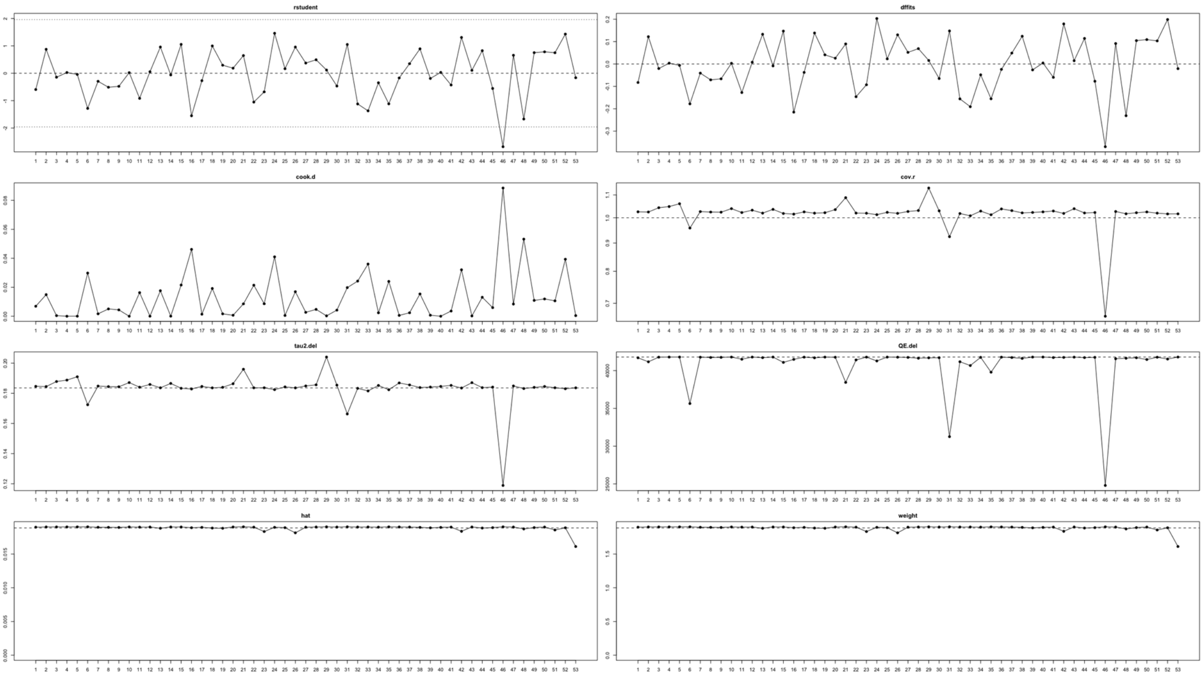

Numbers represent studies which are suggested as below:

Numbers	Study ID	Numbers	Study ID	Numbers	Study ID
1	Abdulateef 2021	19	Feng 2020b	37	Sahni 2021
2	Abdullah 2021	20	Fransis 2020	38	Shu 2020
3	Ahmed 2020	21	Green 2021	39	Sun 2021a
4	Alnajrany 2021	22	Hamdani 2020	40	Sun 2021b
5	Alonso-Castro 2021	23	He 2020	41	Teke 2021
6	Alyami 2020	24	Hellem 2021	42	Tian 2020
7	An 2021	25	Jethani 2021	43	Van der Werf 2021
8	Ashiq 2020	26	Ji 2021	44	Wan 2020
9	Barnes 2021	27	Karbownik 2021	45	Wang 2020
10	Cen 2020	28	Khadka 2021	**46**	**Wong 2021a***
11	Charan 2020	29	Kristiandi 2021	47	Wong 2021b
12	Chen 2020	30	Lam 2021	48	Wu 2020
13	Cheng 2020	31	Lenaerts 2021	49	Yan 2020
14	Chrisinger 2021	32	Ma 2021	50	Zaworski 2020
15	de los Angeles 2020	33	Mamo 2021	51	Zhang 2020
16	Du 2021	34	Nguyen 2021	52	Zhang 2021
17	Erdem 2020	35	Panagiotakos 2021	53	Zhou 2021
18	Feng 2020a	36	Parimala 2021		

*This study was identified to be an outlier.

Supplementary Figure 2. Subgroup analysis based on the study population

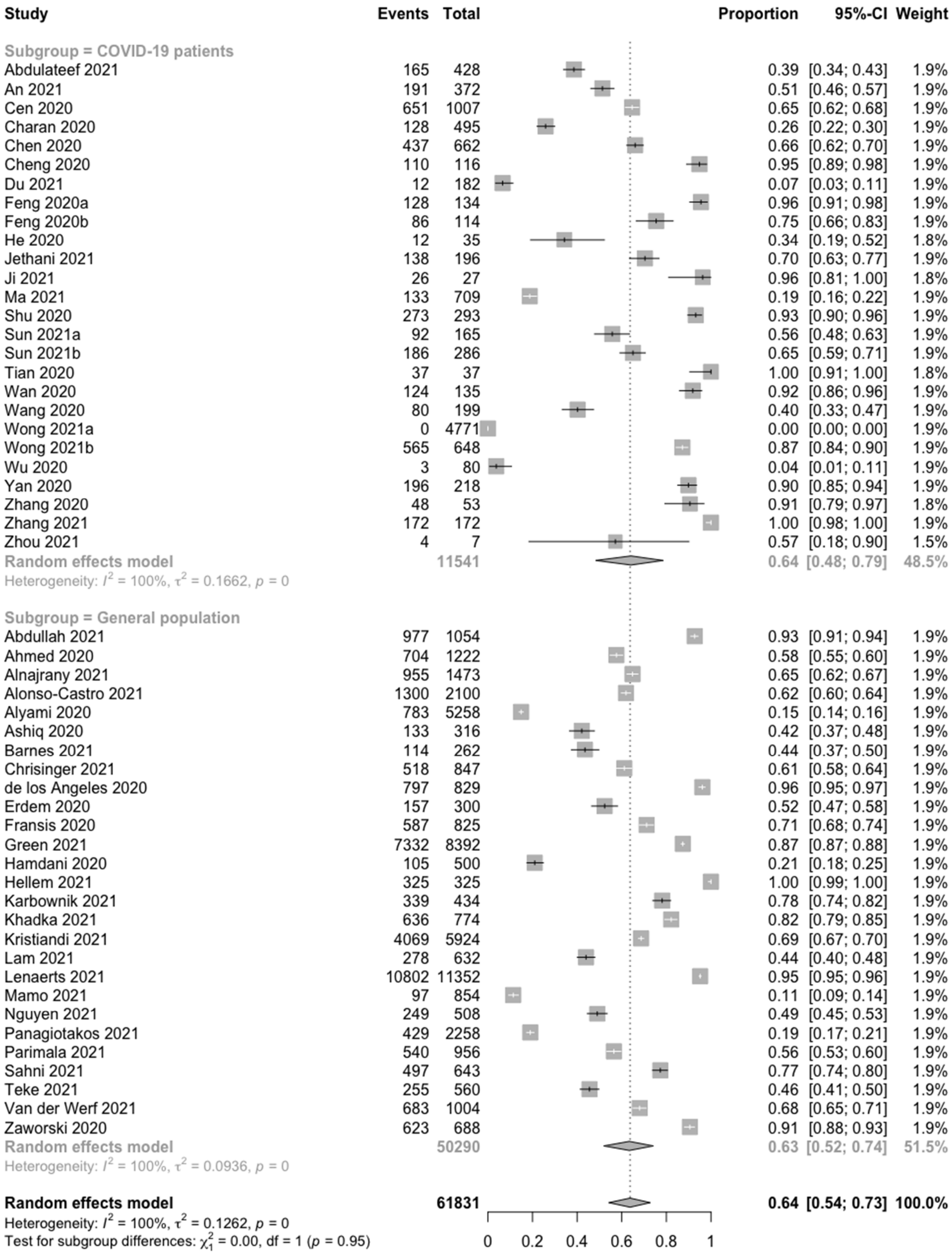

Supplementary Figure 3. Subgroup analysis based on the country

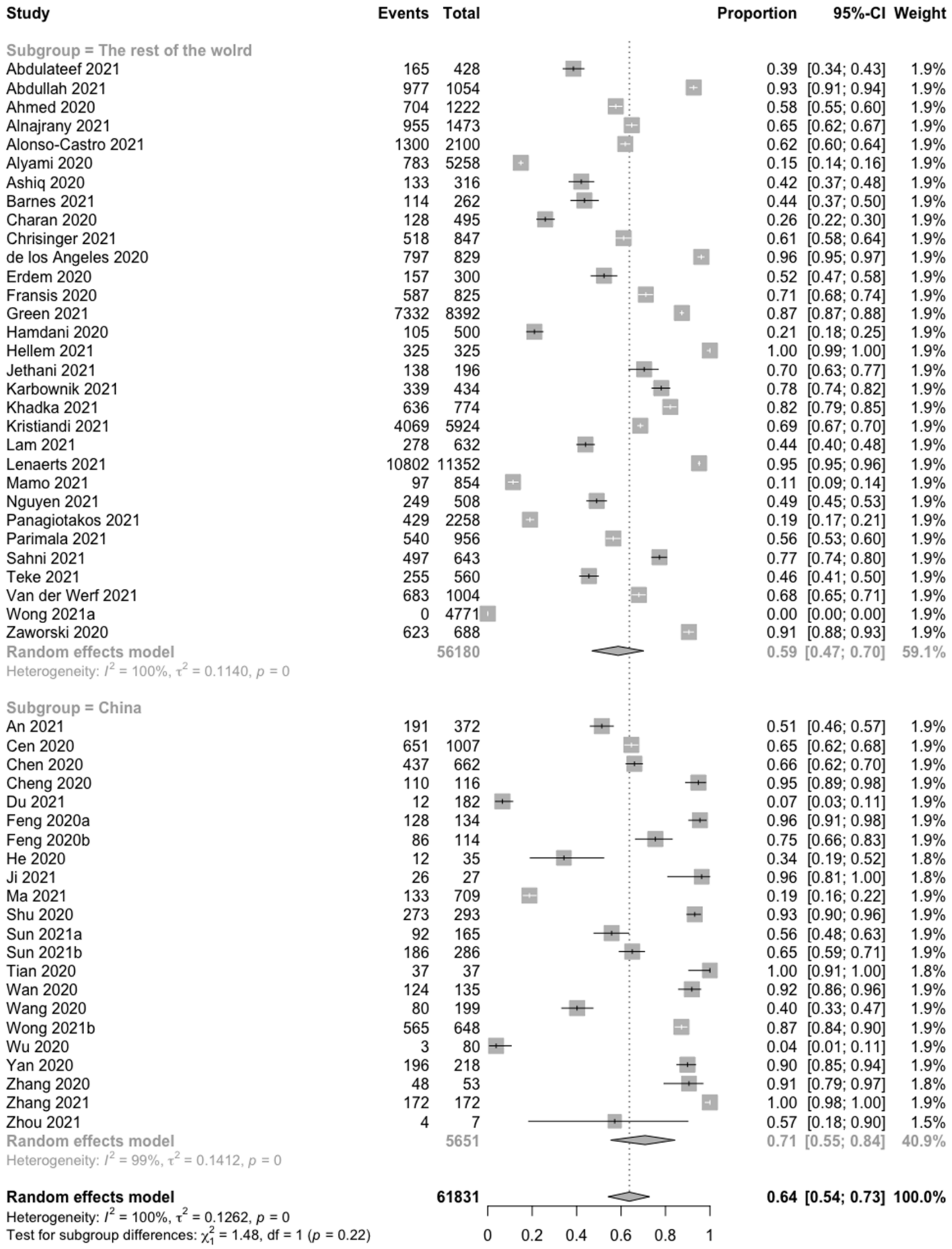

Supplementary Figure 4. Regional distribution of the usage proportion of CAM interventions

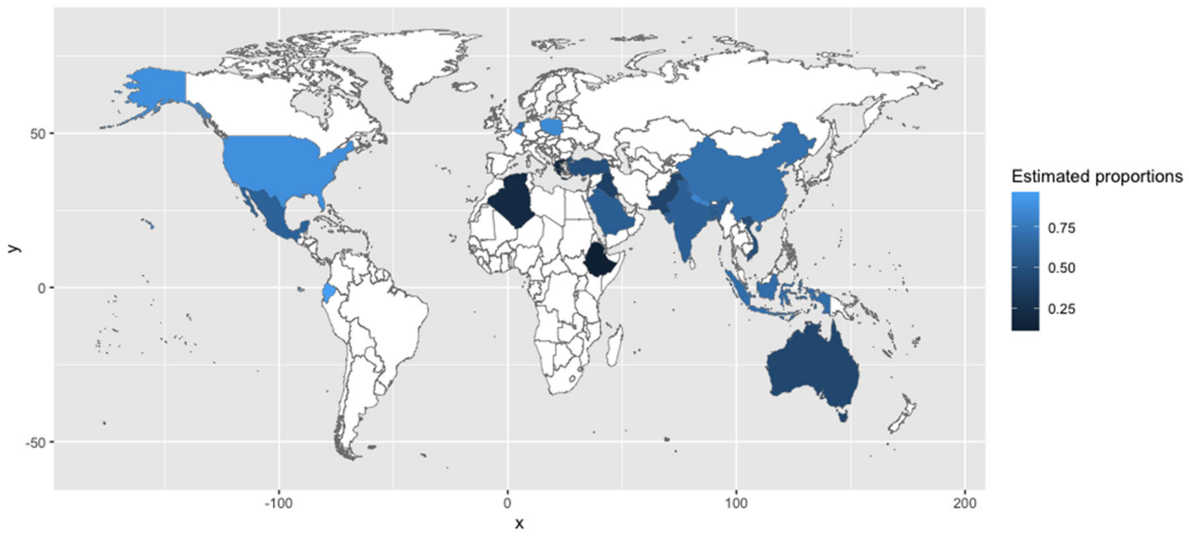

Supplementary Figure 5. Usage prevalence of TCIM

(A) TCM medication

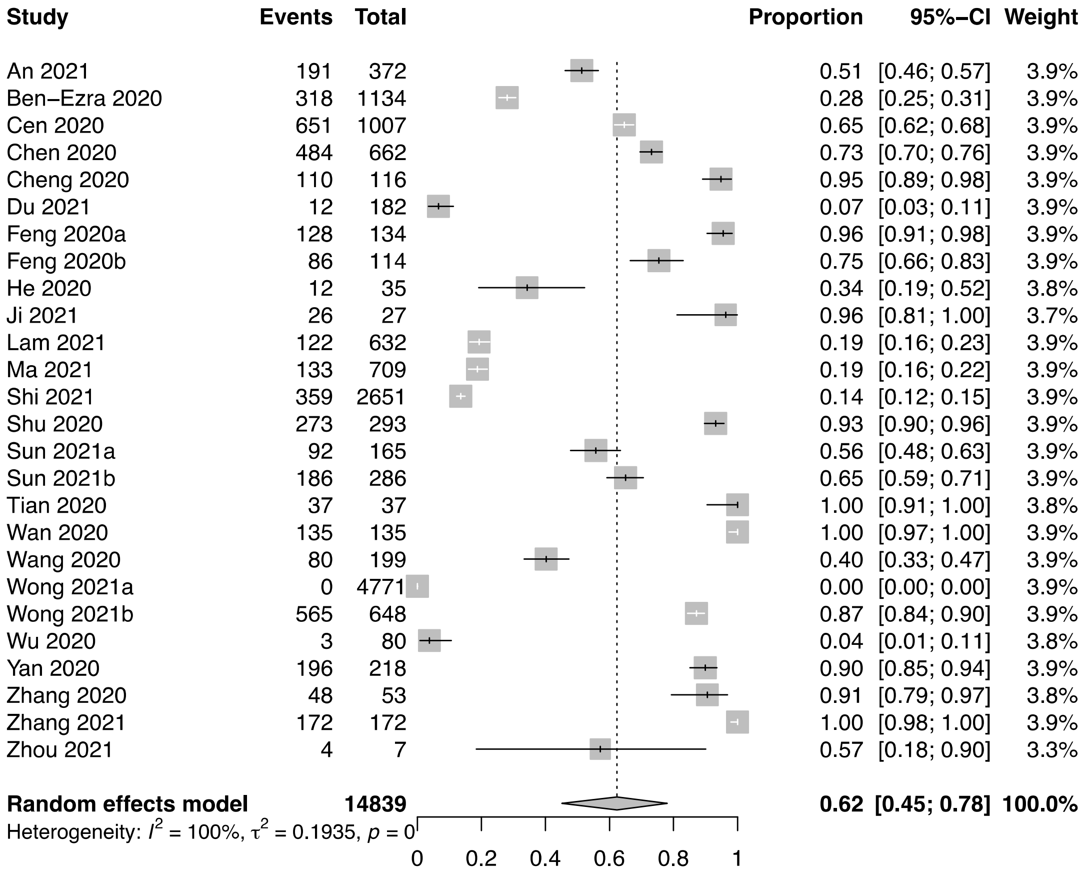

(B) Ayurveda

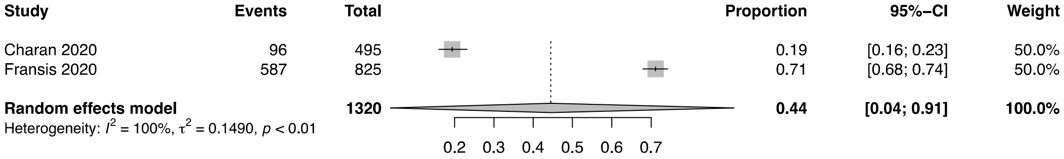

(C) Homeopathy

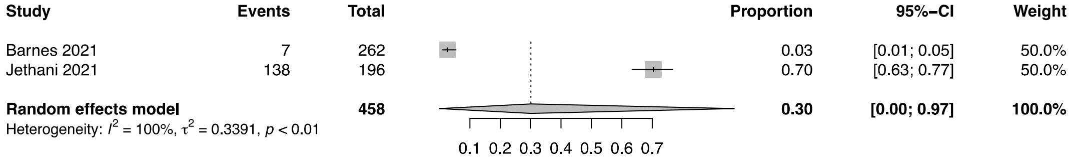

(D) Yoga

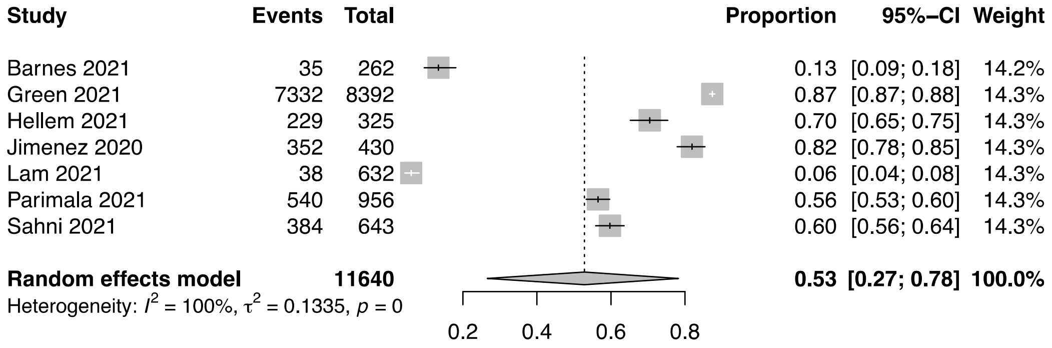

(E) Acupuncture

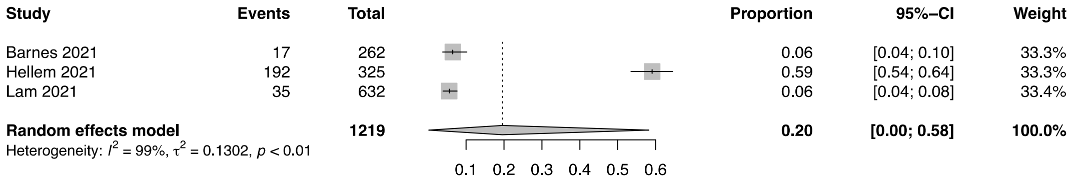

(F) Physical exercise

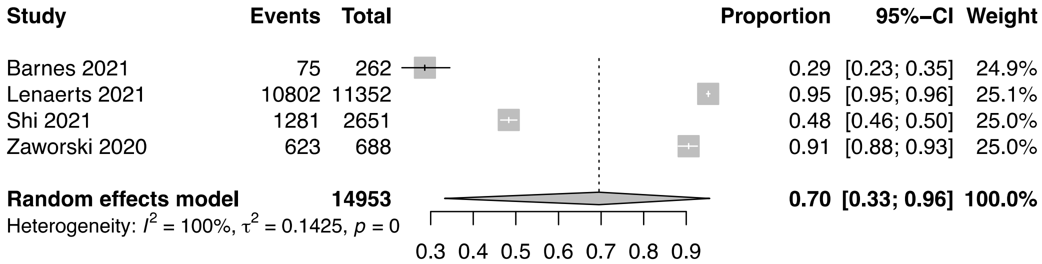

(G) Dietary supplements, herbs, or natural products

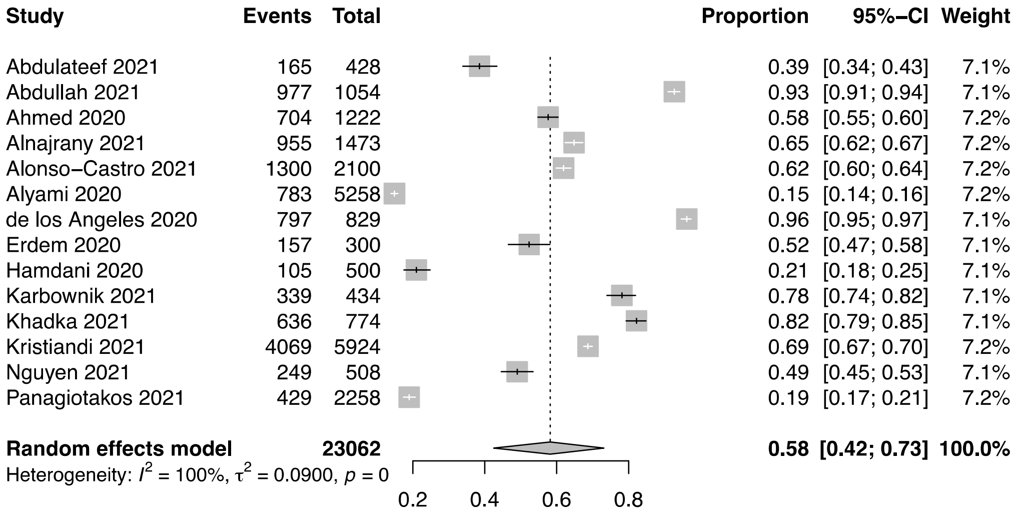

(H) Spiritual therapy

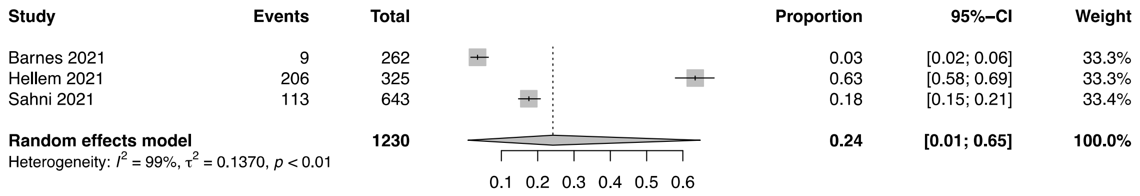

(I) Massage

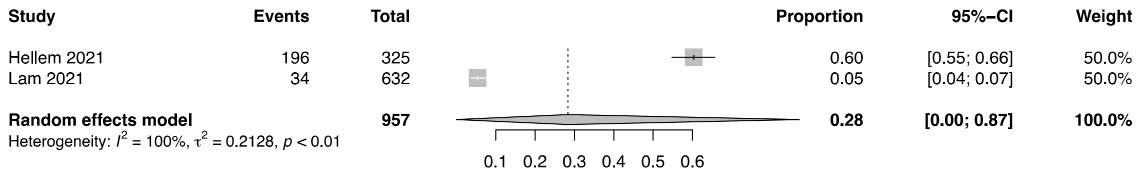

Supplementary Figure 6. Funnel plot for assessing publication bias

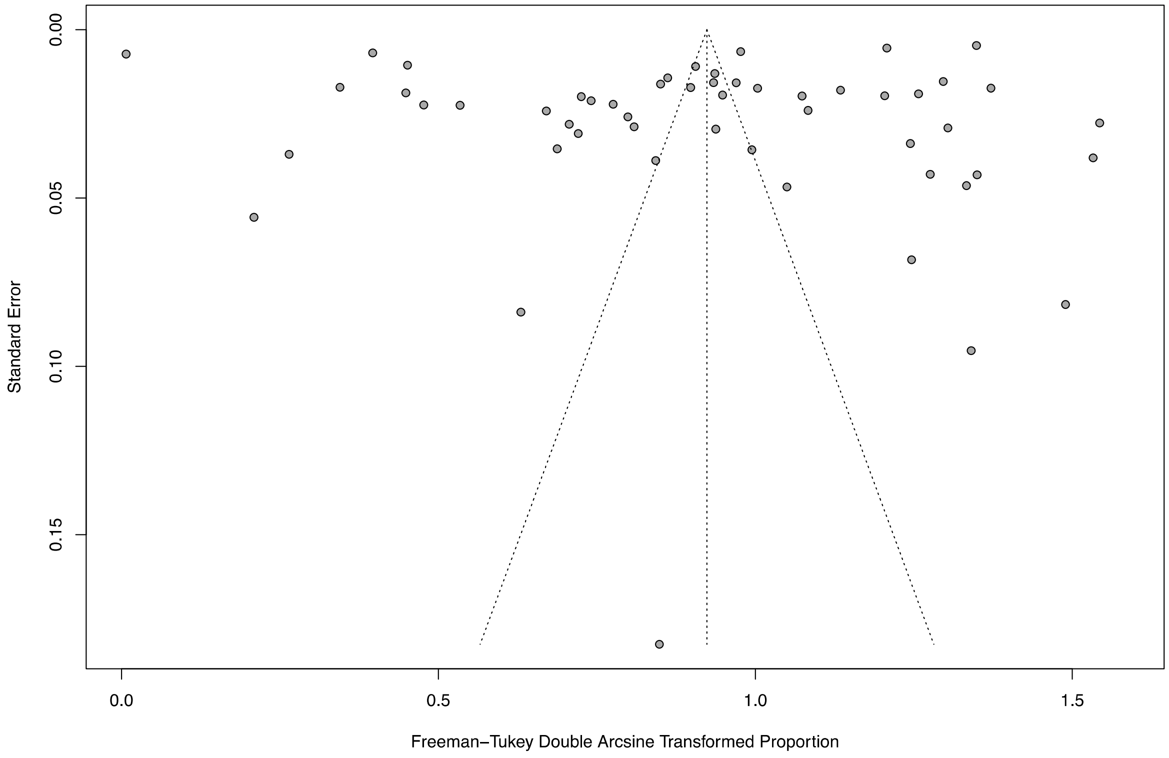

Supplement: Supplementary file 1 [file Data_Sheet_1.pdf]
